# Brain Worms with Cerebrospinal Fluid Eosinophilia

**DOI:** 10.4269/ajtmh.17-0588

**Published:** 2017-12-06

**Authors:** Hui-Ching Shen, Chien-Ming Chao, Cheng-Fang Hsieh

**Affiliations:** 1 Department of Clinical Pathology, Chi Mei Medical Center, Liouying, Tainan, Taiwan;; 2 Department of Intensive Care Medicine, Chi Mei Medical Center, Liouying, Tainan, Taiwan;; 3 Department of Nursing, Min-Hwei College of Health Care Management, Tainan, Taiwan;; 4 Graduate Institute of Medicine, College of Medicine, Kaohsiung Medical University Hospital, Kaohsiung, Taiwan;; 5 Department of Neurology, Kaohsiung Medical University Hospital, Kaohsiung, Taiwan;; 6 Division of Geriatrics and Gerontology, Department of Internal Medicine, Kaohsiung Medical University Hospital, Kaohsiung, Taiwan

A 56-year-old Taiwanese man without any systemic disease suffered from headache for 1 month and general weakness for 3 days. He was sent to the emergency department because of urine incontinence, difficulty in eating and bathing, forgetfulness, and disorientation in time and place for 1 week. He also had head concussion because of a traffic accident about 1 month ago. General soreness, intermittent dizziness, and headache were noted thereafter.

**Figure 1.**
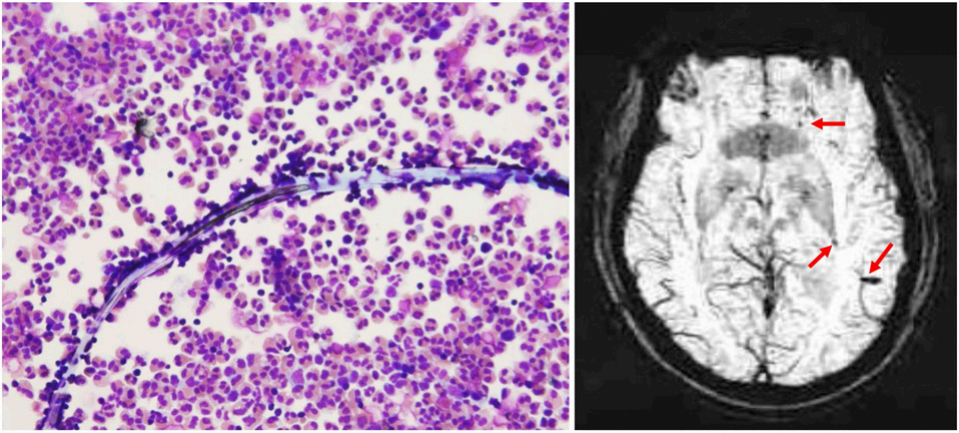
(Left panel) A dead worm with Hemacolor^®^ rapid staining in cerebrospinal fluid (400×). (Right panel) Brain susceptibility weighted imaging magnetic resonance imaging showed multiple microbleeds (arrows) over bilateral cerebral and cerebellar hemisphere. This figure appears in color at www.ajtmh.org.

On admission, vital signs showed afebrile, normal blood pressure, tachypnea, and tachycardia. Physical examination revealed drowsiness, neck stiffness, general weakness, and four limb rigidity. Laboratory data showed a normal white blood cells count, a normal C-reactive protein with eosinophilia (10%), and hyponatremia. Fever up to 38.1°C developed the next day. Chest X-ray reported no active lung lesions and urinalysis was normal. A lumbar puncture demonstrated a normal opening pressure but pleocytosis with eosinophilia (65%). Under the microscope, a suspected dead *Angiostrongylus cantonensis* worm in cerebrospinal fluid (CSF) was found, and the worm head was broken, but its organ was still visible (Figure 1: left panel). His family stated that he had a history of frequent snail catching and eating. A positive enzyme-linked immunosorbent assay for *A. cantonensis* in the CSF was reported later. Brain magnetic resonance imaging (MRI) without contrast revealed multiple microbleeds over bilateral cerebral and cerebellar hemisphere (Figure 1: right panel).

Common symptoms of *A. cantonensis* infection include headache, neck stiffness, and fever, whereas muscle weakness, Brudzinski’s sign/Kernig sign, and hyperesthesia/paresthesia may manifest variably in different case series.^1,2^ Urinary incontinence, cognitive impairment, and parkinsonism as our case are rarely reported. Relative old age and comorbidities such as hyponatremia and head injury may result in different clinical presentations. The detection of young adult worms or larvae in CSF confirms the diagnosis of eosinophilic meningitis due to *A. cantonensis*; however, the detection rate is usually low, with a range from 2% to 11%.^3^

Brain MRI in our patient showed multiple microbleeds over bilateral cerebral and cerebellar hemisphere. Small hemorrhage or hemorrhagic tracts in brain MRI of patients with *A. cantonensis* infection are rarely reported. In patients with eosinophilic meningitis, if subarachnoid hemorrhage or unusual site intracerebral hemorrhage are noted on brain images, gnathostomiasis may be considered as a differential diagnosis.^4^

In summary, it is important for clinicians to consider eosinophilic meningitis and *A. cantonensis* infection when encountering patients with subacute-onset neurologic manifestations, especially a history of snail ingestion, even though afebrile initially.
